# Comparing the effect of bone marrow mono-nuclear cells with mesenchymal stem cells after acute myocardial infarction on improvement of left ventricular function: a meta-analysis of clinical trials

**DOI:** 10.1186/s13287-022-02883-3

**Published:** 2022-05-16

**Authors:** Alireza Hosseinpour, Fatemeh Kheshti, Asma Kazemi, Armin Attar

**Affiliations:** 1 Department of Cardiovascular Medicine, School of Medicine, Shiraz University of Medical Sciences, Shiraz, Iran; 2 Student Research Committee, Shiraz University of Medical Sciences, Shiraz, Iran; 3 Nutrition Research Center, Shiraz University of Medical Sciences, Shiraz, Iran

**Keywords:** Stem cells, Acute myocardial infarction, Bone-marrow mononuclear cells, Mesenchymal stem cells

## Abstract

**Background:**

The effect of transplantation of bone-marrow mononuclear cells (BM-MNCs) and mesenchymal stem cells (MSCs) on ejection fraction (LVEF) has been studied in patients with acute myocardial infarction (AMI) in clinical trials. This raises the question that which type of cell may help improve LVEF better in AMI patients. No meta-analysis of clinical trials has yet addressed this question.

**Methods:**

Electronic databases were searched thoroughly to find eligible trials on the effects of transplantation of BM-MNCs and MSCs in patients with AMI. The primary outcome was improvement in LVEF. Data were synthesized using random-effects meta-analysis. For maximizing the credibility of subgroup analysis, we used the instrument for assessing the Credibility of Effect Modification of Analyses (ICEMAN) for meta-analyses.

**Results:**

A total of 36 trials (26 on BM-MNCs and 10 on MSCs) with 2489 patients (1466 were transplanted [1241 with BM-MNCs and 225 with MSCs] and 1023 as controls) were included. Both types of cells showed significant improvements in ejection fraction in short-term follow-up (BM-MNCs: WMD = 2.13%, 95% CI = 1.23 to 3.04, *p* < 0.001; MSCs: WMD = 3.71%, 95% CI = 2.32 to 5.09, *p* < 0.001), and according to ICEMAN criteria, MSCs are more effective. For selected population of patients who received stem cell transplantation in early course after AMI (less than 11 days), this effect was even more pronounced (BM-MNC: WMD = 3.07%, 95% CI = 1.97 to 4.17, *p* < 0.001, *I*^2^ = 40.7%; MSCs: WMD = 5.65%, 95% CI = 3.47 to 7.84, *p* < 0.001, *I*^2^ = 84.6%).

**Conclusion:**

Our results showed that transplantation of MSCs after AMI might increase LVEF more than BM-MNCs; also, based on ICEMAN, there was likely effect modification between subgroups although uncertainty still remained.

**Supplementary Information:**

The online version contains supplementary material available at 10.1186/s13287-022-02883-3.

## Background

Inadequate blood flow and oxygen supply secondary to formation of thrombus in coronary arteries activate a series of complications leading to myocardial injury, leading to ventricular failure. It is noteworthy that the repair mechanisms following reperfusion in the setting of acute myocardial infarction (AMI) causes irreversible damage to the myocardium by releasing free radicals [1]. On top of that, the infarction and myocardial injury also result in activation of the complement system and production of inflammatory cytokines including interleukin-1 (IL-1), interleukin-6 (IL-6), and tumor necrosis factor-α (TNF-α) [1]. Triggering of chemotactic response and developing an inflammatory microenvironment after infarction eventually induce the process of myocardial tissue degeneration [2]. The contemporary therapeutic guidelines for acute ST elevation myocardial infarction (STEMI) patients only focus on prevention of infarct expansion and have failed to restore the necrotic tissue and cardiomyocytes [3]. Stem cell therapy in patients with AMI was introduced as putative treatment in repairing the damaged myocardium. So far, multipotent stem cells from different sources, such as bone marrow-derived stem cells, circulating progenitor cells, and mesenchymal cells, have been employed for transplantation which can be delivered through different routes [4].

It has been shown that cell therapy of the infarcted myocardium can promote regeneration of the cardiomyocytes and angiogenic capacity by producing different paracrine factors [5]. Among different kinds of cells, bone marrow mononuclear cells (BM-MNCs) and mesenchymal stem cells (MSCs) are the most frequently explored stem cells in trials exploring the potential regenerative effects of stem cells on injured myocardial tissue in AMI patients [4]. Autologous administration of stem cells in humans for assessing their effects on cardiac function in AMI was first appeared in a study at 2001 by Strauer et al. which showed improvement in ejection fraction (LVEF) and reduction in scar size [6]. In the next few years, the bone marrow mononuclear cells were employed in several randomized controlled trials (RCTs) with variable and even contradictory results [7]. Later, investigating the effects of MSC became the subject of some clinical trials. Although a great portion of the trials have provided evidence that application of both BM-MNCs and MSCs results in improvement of myocardial function in the clinical setting, no study has investigated which cellular lineage outperforms the other. Since MSC are more pure stem cells as compared to BM-MNCs, hypothetically it may be expected that they may perform better [8]. However, this hypothesis cannot be confirmed unless it is backed with appropriate well-designed clinical studies and/or meta-analyses. The only available trial directly evaluating this idea, is TAC-HFT trial which is not in the setting of AMI and is conducted in chronic ischemia condition [9]. Although this trial may support this hypothesis, but there are great concerns regarding its limitations. First, the studied sample sizes were too small (19 participants in MSCs group and 19 in BM-MNCs group). Also, the only major factors that were compared between these two groups were reduction in the infarct size and circumferential strain. Thus, we aimed to investigate if MSCs can also perform better in the clinical real-world setting regarding more practical cardiac function parameters such as LVEF, LVEDV, and LVESV which are more frequently used to determine the left ventricular function by conducting a meta-analysis on this topic. This meta-analysis was designed to investigate if administration of either mesenchymal stem cells (MSCs) or bone marrow mononuclear cells (BM-MNCs) in patients with acute ST-segment elevation myocardial infarction has any superiority over the other type of cell regarding the left ventricular function indices (ejection fraction, end-diastolic volume, and end-systolic volume) and major cardiovascular events (rehospitalization for congestive heart failure). Since meta-analyses of clinical trials are of high values and with larger sample sizes, we believe that conducting this meta-analysis is necessary to clarify the answer to the mentioned question.

## Methods

The protocol of this systematic review and meta-analysis was previously registered in PROSPERO (CRD42022296966) and it was synthesized and reported using the methodology recommended by the Cochrane Handbook for Systematic Reviews of Interventions [10].

### Criteria for study selection

#### Types of studies

The potential eligible studies were parallel group controlled clinical trials.

#### Types of participants

All the patients diagnosed with STEMI regardless of age, sex, and baseline echocardiographic indices.

#### Types of interventions

Trials which enrolled the patients with STEMI that were treated with successful primary percutaneous coronary intervention (PCI) and stent implantation or thrombolytics and also those that assigned the participants into a group of intervention which received an injection of either MSCs or BM-MNCs with any delivery route and a control arm which received standard therapy with or without placebo injection were considered for eligibility.

### Types of outcome measures

#### Primary outcomes

Changes in the main echocardiographic indices from baseline including LVEF, left ventricular end-diastolic volume (LVEDV), and left ventricular end-systolic volume (LVESV) were selected as the primary outcome measures of interest. The required follow-up period for measuring the change from baseline values was 4–6 months after the primary measurement of the indices.

#### Secondary outcomes

Hospitalization due to congestive heart failure (CHF) at the longest duration of follow-up was listed as the secondary outcome.

### Search methods for identification of studies

We searched PubMed, Embase, Web of Science, Scopus, and the Cochrane Central Register of Controlled Trials (CENTRAL) to find the relevant English trials using a combination of keywords and controlled vocabulary such as Medical Subject Headings (MeSH). The last search was run on January 10th, 2022, and we applied no restrictions on the time frame. We used the following keywords for this search: “stem cell,” “bone marrow,” “mononuclear cell,” “mesenchymal cell,” myocardial infarction,” and “acute myocardial infarction.” Handsearching of the potentially eligible studies was done for finding other relevant articles.

### Data collection and analysis

#### Selection of studies

**Fig. 1:**
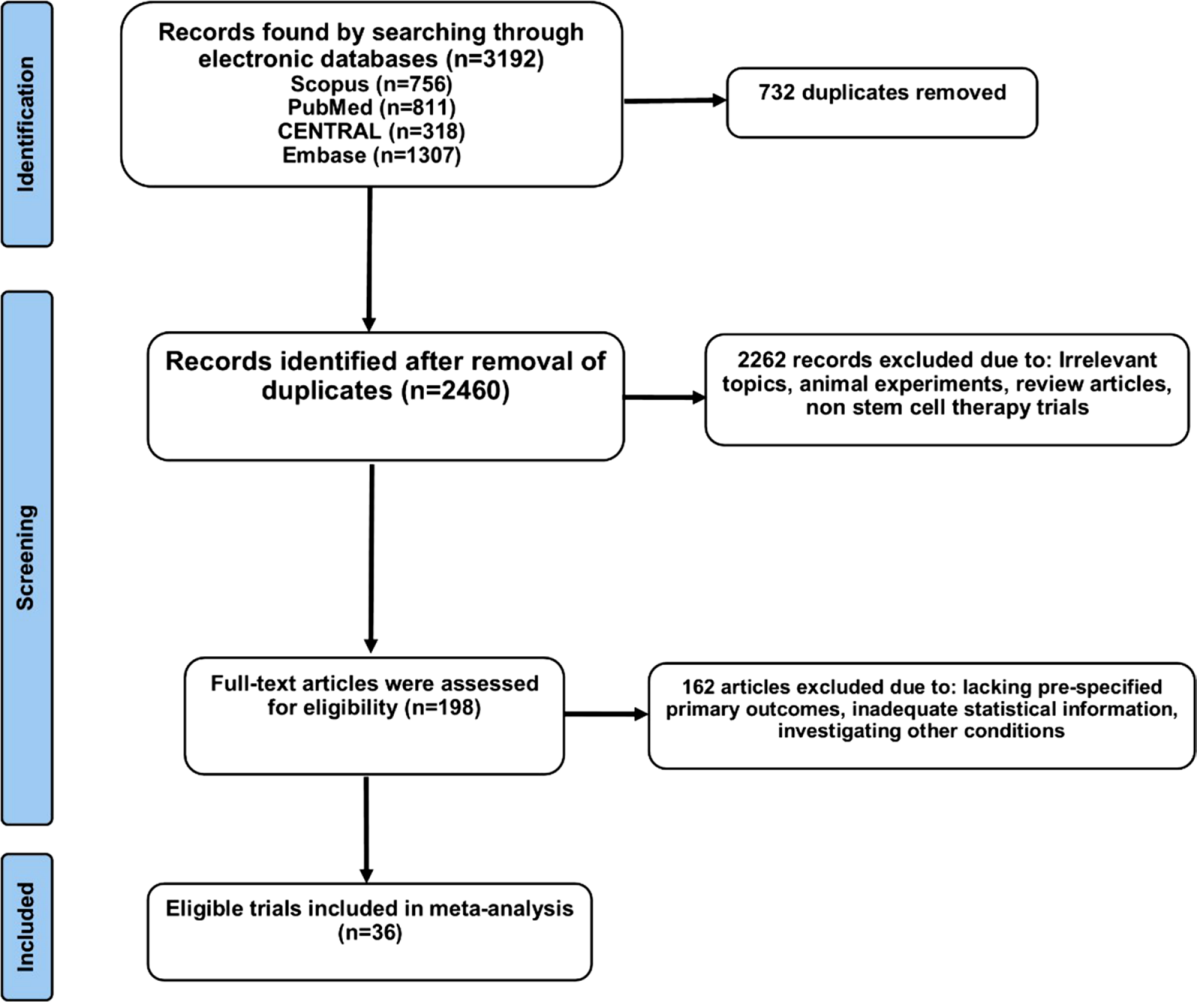
PRISMA flow diagram of the study search and inclusion process

After developing the search strategy, two reviewers (AH and AA) independently screened the abstracts or/and titles of the retrieved records following the removal of duplicate records. We excluded the clearly irrelevant results falling out of the eligibility criteria for this review. Then, the reviewers screened the full text of the remaining articles for final assessment to identify the eligible studies which were in accordance with our pre-specified inclusion and exclusion criteria. In case of any discrepancies, disagreements were resolved through discussion between the two authors. For the present systematic review, we included all the controlled clinical trials which had performed transplantation of either BM-MNCs or MSCs in patients diagnosed with acute myocardial infarction following successful PCI and stent implantation or thrombolytic therapy and compared them to a control arm of acute MI patients treated with standard therapy with or without injection of placebo. The potentially eligible studies were the ones measuring baseline and final values of primary outcomes (LVEF, LVEDV, and LVESV) and the absolute change of final measures from the baseline. A PRISMA flow diagram illustrating the selection process is presented in Fig. 1.

#### Data extraction and management

Two review authors (AH and AA) independently extracted the information and transferred the data to the pre-specified form in Microsoft Excel Spreadsheet Software. The accuracy and consistency of the information was rechecked by both authors and disagreements on data extraction were resolved by discussion between the reviewers. The following information was extracted: characteristics of the included studies (first author, trial name, year of publication, and country of origin), general participant details (baseline demographics and sample size of the intervention and control groups), intervention details (time, dose, and type of injected stem cells), echocardiographic indices (baseline, final, and change from baseline over the follow-up period (with an optimal follow-up of 6 months) of LVEF, LVEDV, and LVESV), the imaging modality used for echocardiographic indices, and major adverse cardiovascular events (rehospitalization for CHF).

#### Quality assessment and risk of bias

The quality appraisal of eligible studies was done by a single author (AH) and rechecked by a second reviewer (AA), using the Cochrane Collaboration’s tool for assessing the risk of bias in randomized trials. The indicators used for risk of bias included selection, performance, detection, attrition, and reporting bias. We reported the risk of bias with Review Manager (RevMan 5.1.7) Software and rated the status of bias in each category as low, unclear, or high risk. If there were any disagreements, we resolved them with discussion.

#### Data synthesis

For our main analysis on echocardiographic indices (LVEF, LVEDV, and LVESV), we expressed continuous data as weighted mean difference (WMD) and 95% confidence interval (95% CI) using the DerSimonian and Laird method [11] with random effects model in which between-study variations were considered. If the absolute change from baseline of indices was not reported, the mean change and its standard deviation (SD) were computed with correlation coefficient formula using the primary and final values of endpoints. Also, for secondary endpoints (rehospitalization for CHF), risk ratio (RR) and its 95% CI were measured as the treatment effect. For assessment of heterogeneity, *I*^2^ and the Cochran’s Q test were used. We performed influence analysis to assess the potential effect of each study on the final results. We carried out all the analyses using Stata software version 13 (StataCorp LP, College Station, TX, USA). Pooled effects with confidence intervals that did not cross the zero line were considered as statistically significant.

#### Subgroup analysis

We conducted a subgroup analysis in this meta-analysis. The eligible trials investigated transplantation of two main lineages of the stem cells (BM-MNCs vs MSCs) for acute MI patients. Thus, we grouped the studies based on the type of cells administered (BM-MNCs group vs. MSCs group) and performed the subgroup analyses on all the outcomes (echocardiographic indices and decompensated heart failure events) to see if there is any significant difference between the two types of cells. To observe the results of studies with more validated method, we conducted a sensitivity analysis; we excluded the studies in which the cells were administered after 11 days after acute MI (injection time ≤ 10 days), the cells were delivered via methods other than intracoronary injection, and the modality used for measuring ventricular indices (LVEF, LVEDV, and LVESV) was other than echocardiography. For interpretation of potential subgroup effects (also called as effect modification), we employed the instrument for assessing the Credibility of Effect Modification of Analyses (ICEMAN) for meta-analyses [12]. This questionnaire comprises 10 questions assessing the credibility of the possible subgroup effect, and for each question, it has four response options (from definitely decreasing the credibility to definitely increasing the credibility). The items generally question if the analysis is between or within trials, if the number of trials are rather large or small, the direction of effect modification has been correctly hypothesized a priori, the random effects model was applied, test for interaction suggests that chance is an unlikely explanation of the apparent effect modification (the between group p value was calculated based on meta-regression), and if a small number of effect modifiers have been used for statistical analysis. The last question rates the overall credibility of subgroup effects based on the number of answers that decreased the credibility for effect modification. The overall assessment could be rated as high, moderate, low, and very low credibility.

## Results

### Description of studies

For this systematic review, preliminary search yielded 3192 records from electronic databases and after removal of 732 duplicate records, abstracts and titles of the remaining ones were screened for eligibility. After full-text screening of 198 studies, 161 articles failed to meet all the inclusion criteria or lacked adequate statistical information. We finally included a total of 36 trials (26 trials with BM-MNCs [13–38] and 10 trials with MSCs [8, 39–47]) for this systematic review. Table 1 summarizes the main characteristics of the included trials. Also, Fig. 1 illustrates the PRISMA flow chart of the assessment process.

#### Study design and settings

Eligible studies for this review included a total of thirty-six trials (26 trials with BM-MNCs and 10 trials with MSCs). All the trials were parallel-group randomized controlled designed except one study [46] on MSCs which was non-randomized. Nineteen trials were multi-center studies [8, 13, 14, 18, 21, 25, 26, 29–32, 34, 37, 39–41, 44, 45, 47], eleven single-center trials [15–17, 19, 20, 22, 23, 27, 33, 38, 43], and six studies were unknown regarding the status of the centers [9]. As trials were using different routes of delivery, and transplantation time intervals and these parameters would affect the outcomes, we selected a population of trials whose route of delivery was intracoronary and the transplantation time was below 11 days and performed a subgroup analysis for them in all variables.

#### Participants

A total of 2489 patients diagnosed with STEMI were included in the trials, of whom 1466 received transplantation of stem cells (1241 with BM-MNCs and 225 with MSCs) and 1023 participants were included in the control group which had received either placebo injection or standard therapy for AMI. The primary intervention for all participants was percutaneous coronary intervention (PCI) (10 trials PCI only on LAD [14, 15, 17, 27, 30, 32, 33, 38, 42, 43]) in all trials, except two studies [25, 26] that performed fibrinolysis with fibrinolytics and one trial [29] used either PCI or fibrinolytics as their first intervention.

#### Interventions and comparators

Stem cell transplantation (BM-MNCs or MSCs) was performed for the intervention group and the control group received either an injection of placebo or standard care based on the current guidelines. The route of stem cell delivery was intracoronary injection for the majority of trials, except one with intramyocardial [46], one with intravenous [8] injection, and one trial delivering the cells via retrograde intravenous coronary route in one of the intervention groups [26]. Some trials divided the intervention groups based on the time interval for injection [20, 31], cell dosage [24, 37], condition of cells (hypoxia vs. normoxia [19] and irradiated vs. non-irradiated cells [37]), and single vs. repeated injections [38]. Bone-marrow aspiration and injection of placebo were performed for control groups as a sham procedure in several studies [13, 16, 22, 25, 32–34, 36], whereas in some trials any sham procedure or placebo injection was avoided [14, 17–19, 23, 24, 26–31, 35, 40, 43, 44, 46, 47]. A few trials avoided bone-marrow aspiration for the control group but administered a dose of placebo injection for them [15, 20, 21, 37, 38]. Bone-marrow aspirate was collected from unrelated healthy donors in three trials [8, 39, 45]. In MSC trials, the sources for stem cells other than bone marrow were derived from the umbilical cords of healthy donors [41] and liposuction from the periumbilical region [42].

#### Risk of bias in included studies

For assessing the risk of bias in eligible studies, we investigated selection, performance, detection, attrition, and reporting bias. We evaluated if the studies had a clear method for random sequence generation stated in the manuscript or its protocol. Twenty-four studies presented an adequate method for random sequence generation [13–19, 21, 22, 24–27, 29, 31–34, 37–41, 47]. The mentioned methods include randomization list [13, 14], random numbers between 0 and 1 [15], sequentially numbered sealed envelopes [16, 22, 24, 31], permuted block randomization [17, 18], randomization number table [19], computer-generated block randomization [21, 25, 39, 41], blocks by means of sealed envelopes [26], uneven vs. even numbers [27], central telephone system and blocking [29], random or sequential numbers [40, 47], interactive web-based randomization session using randomly selected block sizes of 6 or 9 stratified by center [32], randomization algorithm developed by biostatistician [33], and computer- generated random number sequence using sequentially numbered sealed opaque envelopes [38]. Twelve studies lacked a clear method for randomization and were classified as unclear or high risk for random sequence generation [8, 20, 23, 28, 30, 35, 36, 42–46]. Also, allocation was concealed properly and risk of selection bias due to allocation concealment was rated as low in eighteen trials [8, 13–16, 19–23, 32–34, 38–42]. Masking was not done for either study groups or personnel or both of them in nine trials [17, 18, 20, 26, 29–31, 41, 44], and nine other studies were unknown regarding the blinding process [14, 15, 23, 24, 27, 28, 35, 38, 46]. Outcome assessors were blinded to treatment allocation in the majority of trials, except six studies which had unclear or high risk [17, 20, 22, 28, 46, 47]. Seven trials were rated as high risk for attrition bias as they had high rates of withdrawals, or withdrawals were unequal between the study groups [20, 28–31, 37, 44]. Also, three trials had unclear risk since the rate of withdrawals was not stated clearly [23, 26, 27]. For selective reporting or reporting bias, one study was at high risk [26] and six studies had unclear risk [16, 17, 23, 35, 36, 38]. Summary of the risk of bias is presented in Additional file 1.

#### Effects of interventions

For this meta-analysis, the primary goal was to compare two types of stem cells (BM-MNCs and MSCs) used for patients following acute myocardial infarction regarding their effect on left ventricular echocardiographic indices (LVEF, LVEDV, and LVESV) and rehospitalization for heart failure. For providing an easier way for interpretation of subgroup difference between the two types of cells, we employed ICEMAN tool for our analyses. The summary of ICEMAN instrument made for this review is shown in Table 2.

#### Left ventricular ejection fraction

**Fig. 2:**
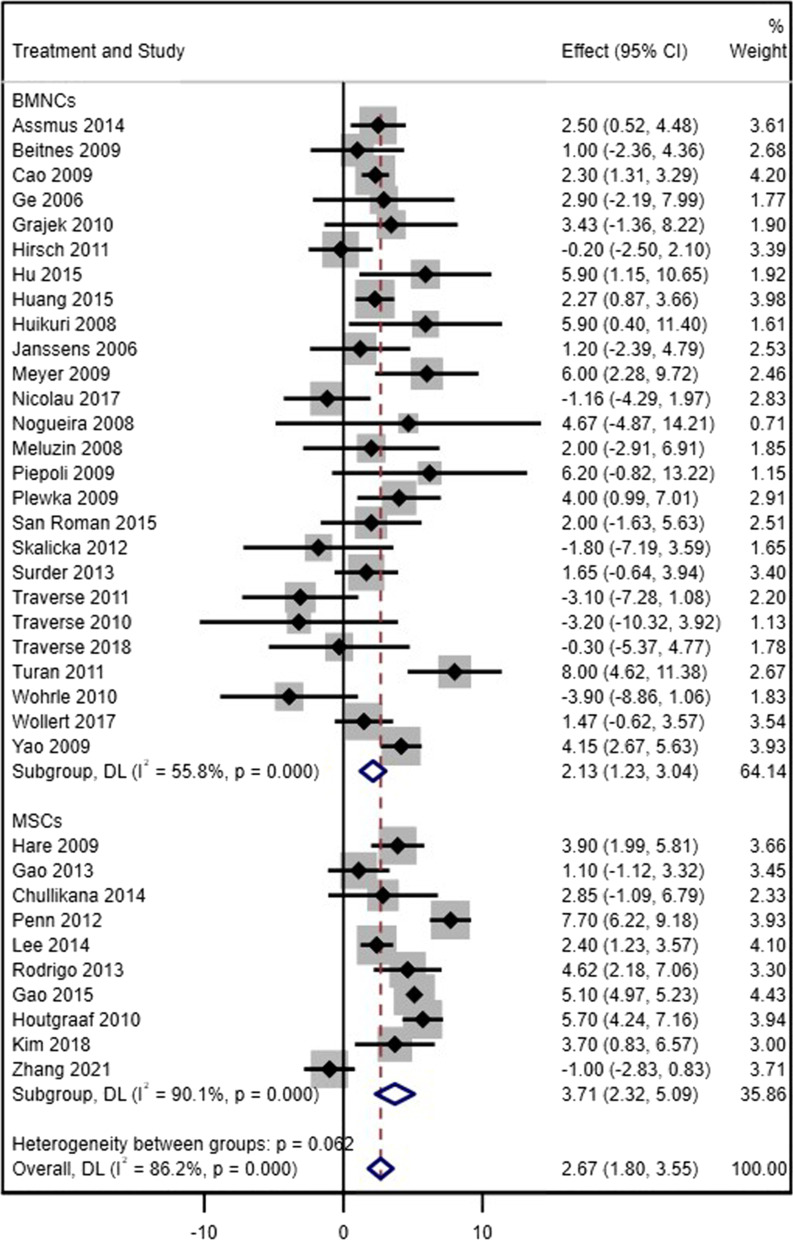
Forest plot of the effect sizes of changes in LVEF from baseline during the short-term follow-up (4–6 months) in acute MI patients who received injection of either BM-MNCs or MSCs compared to the control group who received standard therapy with or without placebo injection

Thirty-six studies (26 studies on BM-MNCs and 10 studies on MSCs) were included for analysis of change in LVEF from baseline to 4–6 months of follow-up. A significant improvement in LVEF was seen in both BM-MNC trials (WMD = 2.13%, 95% CI = 1.23 to 3.04, *p* < 0.001, *I*^2^ = 57.3%) and MSC trials (WMD = 3.71%, 95% CI = 2.32 to 5.09, *p* < 0.001, *I*^2^ = 90.1%) (Fig. 2). According to ICEMAN criteria, the difference between BM-MNCs and MSCs was significant. For sensitivity analysis (intracoronary injection, modality for ventricular indices was echocardiography, and the time from myocardial infarction to stem cell injection was before 11 days), nine BM-MNC trials and three MSC trials were included, and there was evidence of a significant change for both types of cells (BM-MNC: WMD = 3.07%, 95% CI = 1.97 to 4.17, *p* < 0.001, *I*^2^ = 40.7%; MSCs: WMD = 5.65%, 95% CI = 3.47 to 7.84, *p* < 0.001, *I*^2^ = 84.6%) (Additional file 2).

#### Left ventricular end-diastolic volume

**Fig. 3:**
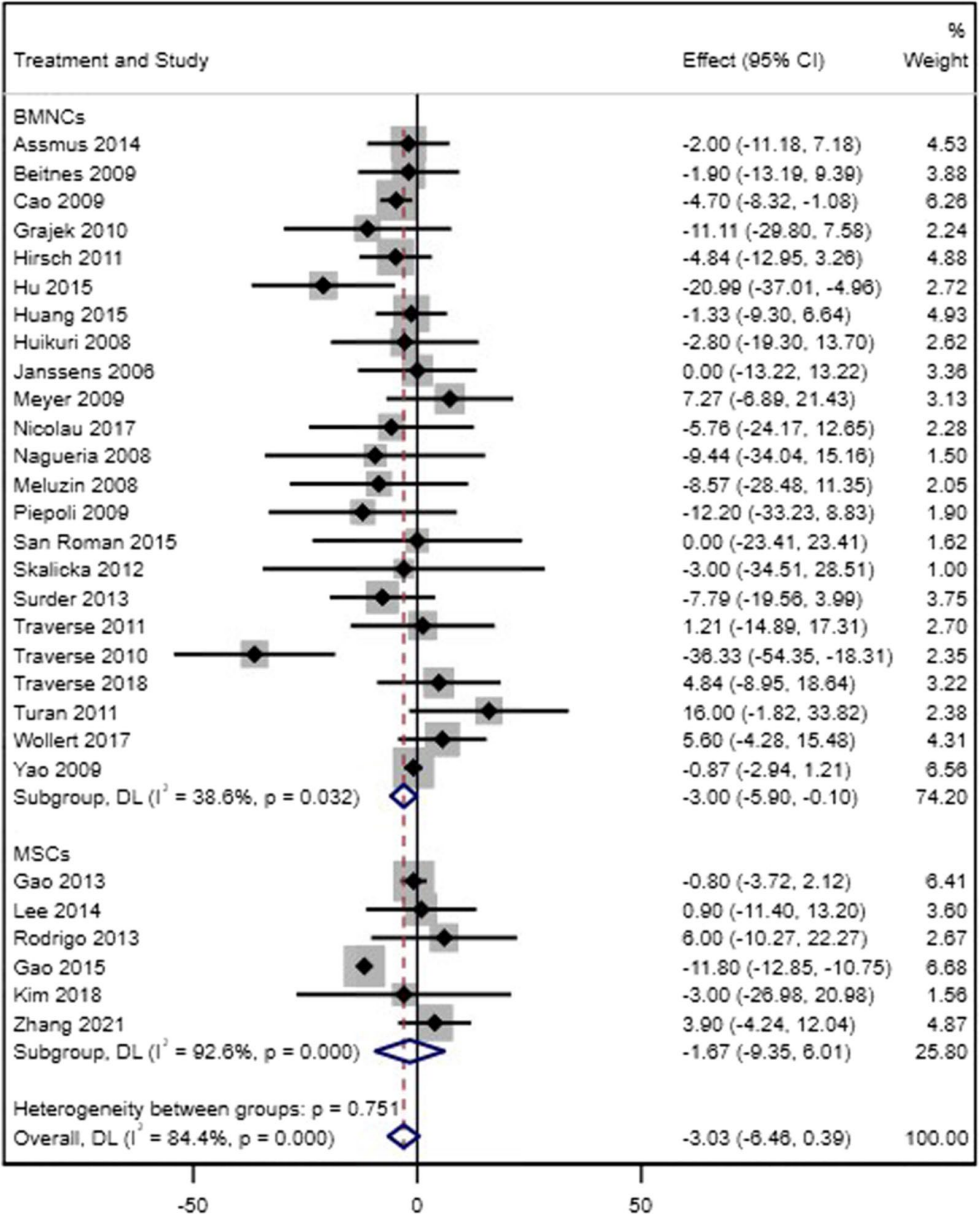
Forest plot of LVEDV changes in acute MI patients who received either standard therapy (with or without placebo injection) or autologous injection of stem cells based on the type of cell (BM-MNCs or MSCs)

Twenty-three BM-MNC trials and six MSC trials reported changes in LVEDV during 4–6 months after stem cell therapy. There was evidence of a significant change in LVEDV following BM-MNC therapy (WMD = − 3.00, 95% CI = − 5.90 to − 0.10, *p* = 0.043, *I*^2^ = 18.8%), but no difference in the level of LVEDV was observed after transplantation of MSCs (WMD = − 1.67, 95% CI = − 9.35 to 6.01, *p* = 0.671, *I*^2^ = 92.6%) (Fig. 3). There was no evidence for a difference between the two types of cells regarding LVEDV values (*p* = 0.84). Eight BM-MNC trials and only one MSC trial were available for sensitivity analysis, and there was no significant change in LVEDV in BM-MNC trials (WMD = − 4.25, 95% CI = − 9.48 to 0.98, *p* = 0.111, *I*^2^ = 39.7%), but a significant decrease in LVEDV was observed in MSC trial (WMD = − 11.80, 95% CI = − 12.85 to − 10.75) (Additional file 3). There was a significant difference between MSC trial and BM-MNC trials in sensitivity analysis (*p* = 0.007).

#### Left ventricular end-systolic volume

**Fig. 4:**
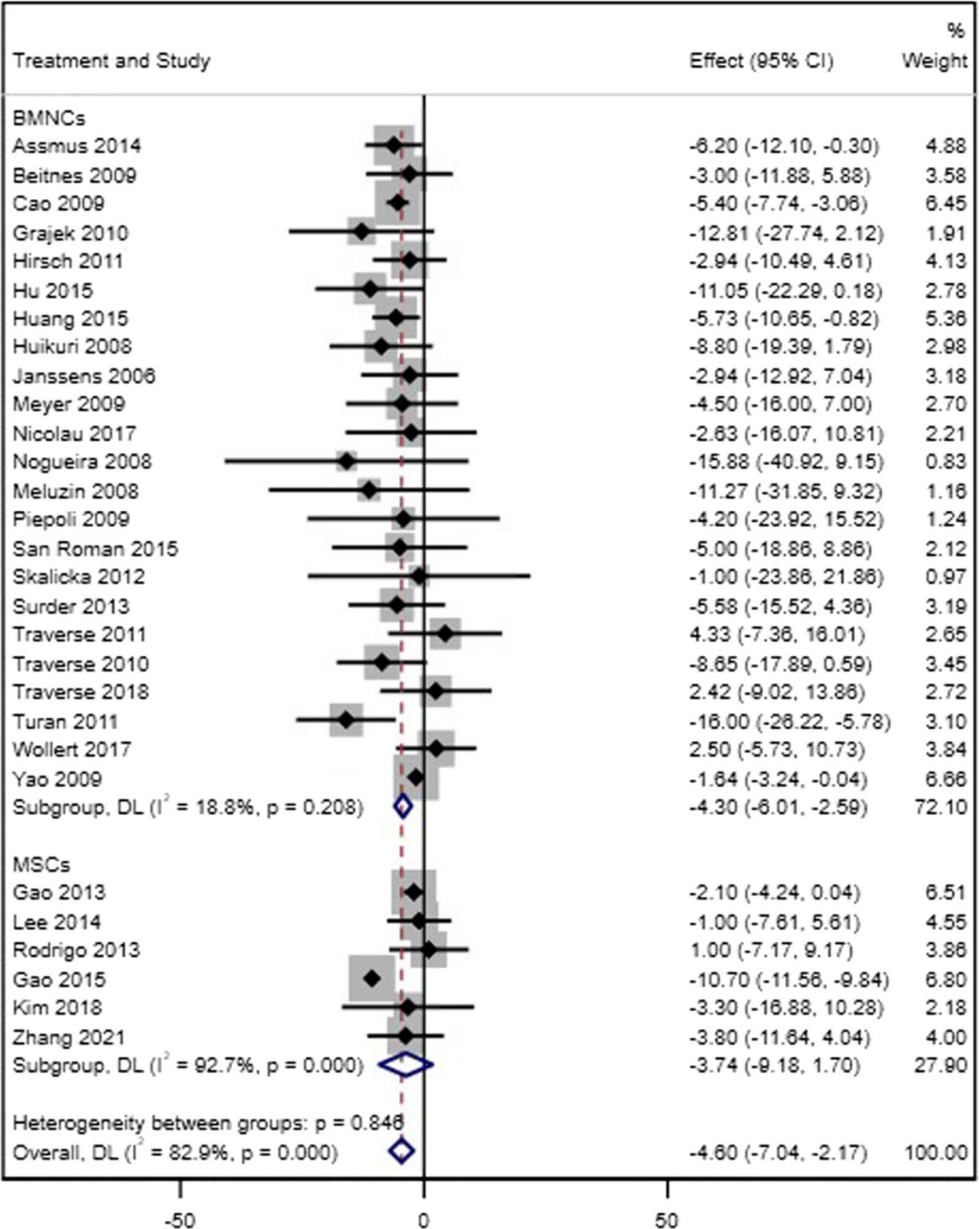
Forest plot of comparison of changes in LVESV over the follow-up period in patients with acute MI who received stem cell therapy based on the type of cells (BM-MNCs or MSCs) compared to the control group

For LVESV, twenty-three trials for BM-MNCs and six trials for MSCs were assessed. BM-MNC therapy resulted in a significant decrease in LVESV (WMD = − 4.30, 95% CI = − 6.01 to − 2.59, *p* < 0.001, *I*^2^ = 18.8%), whereas in MSC trials there was no significant change in the level of LVESV (WMD = − 3.74, 95% CI = − 9.18 to 1.70, *p* = 0.178, *I*^2^ = 92.7%) (Fig. 4). In sensitivity analysis, seven BM-MNC trials and one MSC trial were included, and there was a significant decrease in LVESV for both types of cells (BM-MNC: WMD = − 6.99, 95% CI = − 9.95 to − 4.03, *p* < 0.001, *I*^2^ = 17.1%; MSC: WMD = − 10.70, 95% CI = − 11.56 to − 9.84) (Additional file 4). There was no evidence of a significant difference between the two groups in both analyses (*p* = 0.92, *p* = 0.15, respectively).

#### Hospitalization for congestive heart failure

**Fig. 5:**
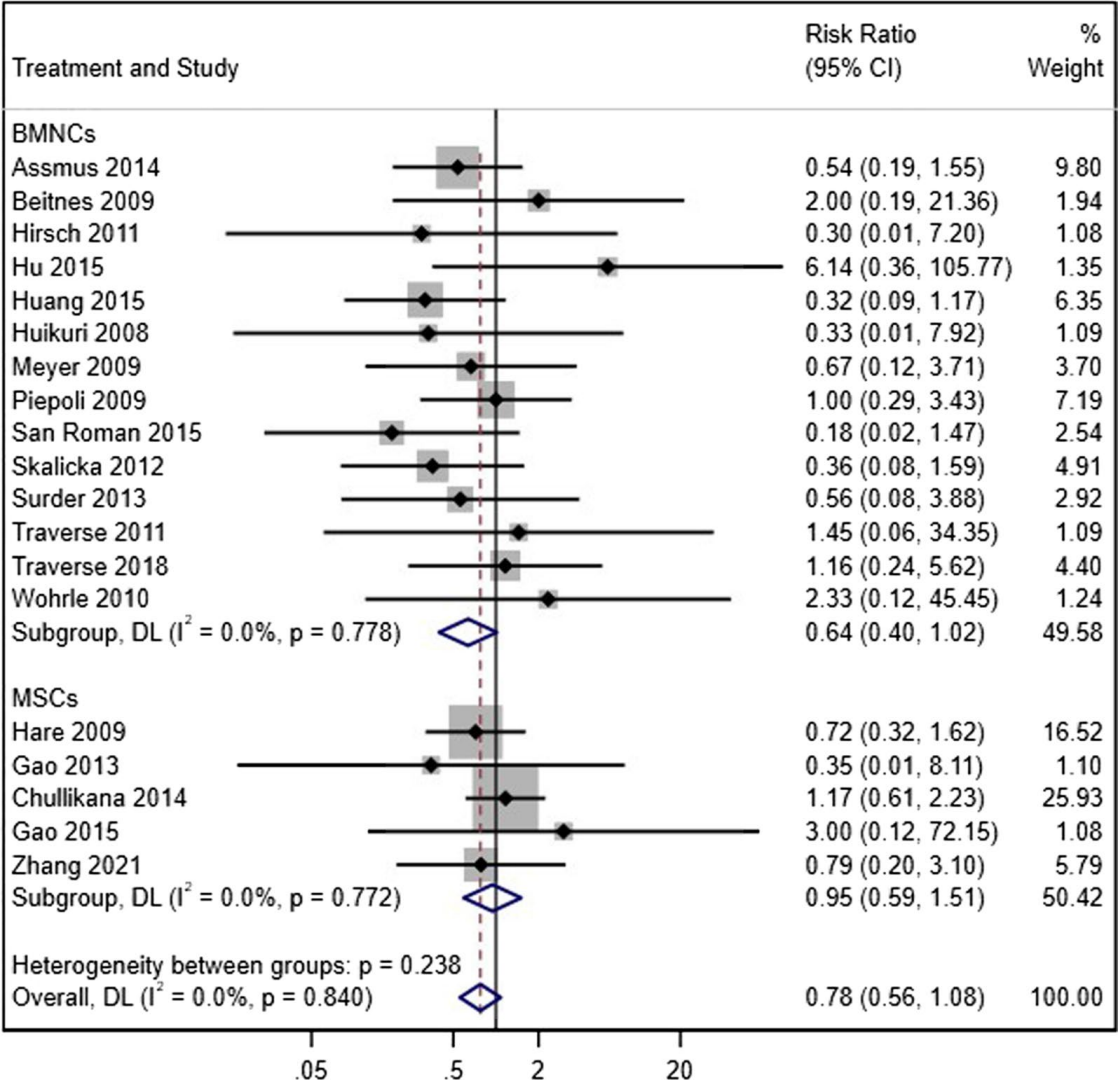
Forest plot of comparison of the rate of hospitalization due to heart failure in acute MI patients who received stem cell therapy (BM-MNCs or MSCs) compared to the control group who received standard therapy with or without placebo injection

The rate of hospitalization for decompensated heart failure at the longest duration of follow-up was reported in fourteen BM-MNC trials and five MSC trials. For both groups, the rate of hospitalization did not differ significantly compared to the controls (BM-MNCs: RR = 0.64, 95% CI = 0.40 to 1.02, *p* = 0.058, *I*^2^ = 0.0%; MSCs: RR = 0.95, 95% CI = 0.59 to 1.51, *p* = 0.813, *I*^2^ = 0.0%) (Fig. 5), and for between BM-MNC and MSC group, no difference was observed (*p* = 0.26).

## Discussion

In the present study, by including 36 trials and 2489 patients, we found that MSC therapy improved LVEF more effectively as compared to BM-MNCs after AMI (3.67% vs. 2.13%); if this therapy was performed within the first 10 days after AMI, its effect might increase (5.65% vs. 3.07%). To the best of our knowledge, this meta-analysis is the first study conducted to compare the effect of two types of cell therapy in the patients with AMI.

Effects of stem cell therapy after acute myocardial infarction have been widely studied. Bone marrow-derived mononuclear cells (BM-MNCs) and mesenchymal stem cells (MSCs) are two of the most common and accessible types of stem cells used in clinical studies, both types of which have shown to improve the ventricular indices, specifically ejection fraction [48, 49]. Results from a meta-analysis showed that injection of BM-MNCs in patients diagnosed with STEMI improved the ejection fraction by 2.21% in the short-term follow-up period (≤ 6 months) and 3.68% in the long-term follow-up (≥ 1 year) [50]. In another meta-analysis on 956 patients with AMI treated with MSCs, LVEF increased by 3.78%, and injection time of ≤ 7 days resulted in a better increase when compared to later time intervals (5.74% vs. 2.35%, respectively) [51]. Although both BM-MNCs and MSCs appeared to be effective in patients with AMI, no study has investigated if any type can outperform the other one and be more efficacious for reconstructing the infarcted area in acute myocardial infarction. The only study that compared MSC with BM-MNCs directly was the TAC-HFT which was conducted in patients with chronic MI-induced ischemic cardiomyopathy and not AMI. In TAC-HFT trial, the investigators found that transendocardial injection of MSCs in patients with chronic MI-induced ischemic cardiomyopathy resulted in a significant increase of viable tissue mass (8.4%) despite the fact that transendocardial injection of BM-MNCs did not change the viable mass significantly (3.4%) during a 12-month follow-up. Also, it was shown that MSCs appeared to decrease the scar size as a percentage of LV mass significantly (18.9%); in contrast, BM-MNC therapy did not cause a significant increase when compared within groups (− 7.0%) [9].

Based on the results of TAC-HFT randomized trial, it can be assumed that MSCs might be more effective than BM-MNCs in improving the function of the left ventricle after AMI as well. In the subgroup analysis, we observed a significant increase of 2.13% in LVEF following transplantation of BM-MNCs, whereas there was a significant improvement of 3.71% for LVEF in AMI patients transplanted with MSCs (MSCs improved LVEF by about 1.6% more than BM-MNCs). For better interpretation of subgroup analysis between the two types of cells, we employed the ICEMAN questionnaire [12] in our review, as shown in Table 2. According to ICEMAN questions, there was only one question that decreased the credibility of subgroup difference (Q5: Does a test for interaction suggest that chance is an unlikely explanation of the apparent effect modification?) for LVEF. The overall interpretation for subgroup difference indicated that there is a possible effect modification when BM-MNCs and MSCs are compared. For sensitivity analysis, we excluded studies in which the route of delivering stem cells was not intracoronary, time interval between diagnosis of AMI and stem cell therapy was more than 10 days, and the modality used for assessing LV function was not echocardiography. LVEF was improved by 5.65% in the mesenchymal group and 3.07% by the mononuclear group. As hypothesized in a prior protocol, the direction of subgroup difference in both analyses provided evidence of superiority of MSCs over BM-MNCs and ICEMAN method showed that this subgroup difference is most likely credible, but uncertainty still remains. For the other two echocardiographic indices (LVEDV and LVESV), BM-MNCs showed better results than MSCs, but their differences did not reach a significant level; in sensitivity analyses, only one trial was included for MSCs and based on ICEMAN, a high level of uncertainty was assumed. Transplantation of BM-MNCs has been numerously employed in different trials in AMI patients although trials on MSCs are less frequently studied since MSCs have entered clinical trials more recently. One explanation regarding the better effects of BM-MNCs on LVEDV and LVESV is that since fewer trials have studied MSCs compared to BM-MNCs, the smaller sample size of MSC trials have led to contradictory results. One point that can support this explanation is that according to our sensitivity analyses of LVEDV and LVESV, which we compared trials with similar modalities and route and time of injection, MSCs were able to yield better results regarding LVEDV and also LVESV although since only one MSC trial was included in the analysis, no definite effect can be assumed (based on ICEMAN) and conduction of future MSC trials are crucial to confirm the possible outperformance of MSCs compared to BM-MNCs. Also, for hospitalization for HF both groups did not show a significant difference compared to their control group, and there seemed to be no effect modification based on ICEMAN.

Since ventricular dysfunction and the subsequent decompensated heart failure carry the most cardiac-related precipitating factor for mortality of AMI patients [52], preventing ventricular dysfunction is of great importance in these patients. As utilization of the stem cells in acute myocardial infarction is becoming more established in clinical trials, a great endeavor should be made to find the most effective type of cell for AMI patients. In the present systematic review and meta-analysis, we found that MSCs improve ejection fraction more than BM-MNCs although a level of uncertainty should be reminded. For other outcomes in our review (LVEDV, LVESV, and hospitalization), it is noteworthy that our findings were equivocal since for LVEDV and LVESV mononuclear cells had better results although their difference remained insignificant, and for hospitalization both of stem cells did not change the hospitalization rate due to CHF.

This study also had several limitations. We included a non-randomized clinical trial for MSCs since trials of MSCs included a total of 451 patients, which were limited compared to BM-MNCs that had a total of 2038 patients. Different modalities were used for measuring ventricular indices such as echocardiography, CMR, SPECT, and LV angiography, and this can cause some differences in interpretations. Another issue was that the baseline ejection fractions for patients were different and ranged from 33 to 62, and this can significantly change the results of trials.

## Conclusion

This meta-analysis provided evidence that both BM-MNCs and MSCs enhanced ventricular function by improving LVEF and MSCs appeared to be superior to BM-MNCs regarding the improvement of ejection fraction although these results cannot be interpreted without a level of uncertainty. Other ventricular parameters including LVEDV and LVESV and the rate of hospitalization for heart failure had equivocal results.

**Table 1:** Characteristics of the included trials

Study	Country	Type of cell	Sample size		Mean age		Male%		Baseline LVEF (%)		Injection time (days)	Modality
			Inv	Cont	Inv	Cont	Inv	Cont	Inv	Cont		
Assmus [13]	Germany	BM-MNCs	101	103	55 ± 11	57 ± 11	82	82	47.5 ± 10	46.7 ± 10.3	4.4 ± 1.3	LV angiography
Beitnes [14]	Norway	BM-MNCs	50	50	58.1 ± 8.5	56.7 ± 9.6	84	84	45.7 ± 9.4	46.9 ± 9.6	4–8	Echo/CMR
Cao [15]	China	BM-MNCs	41	45	50.7 ± 1.1	51.0 ± 1.0	95	93	39 ± 3	38.6 ± 3	7	Echo/SPECT
Ge [16]	China	BM-MNCs	10	10	58 ± 11	59 ± 8	80	100	53.8 ± 9.2	58.2 ± 7.5	0–1	Echo/SPECT
Grajek [17]	Poland	BM-MNCs	31	14	49.9 ± 8.4	50.9 ± 9.3	87	86	50.32 ± 9.8	50.84 ± 11.97	4–5	Echo/SPECT
Hirsch [18]	Netherlands	BM-MNCs	69	65	56 ± 9	55 ± 10	84	86	43.7 ± 9	42.4 ± 8.3	8	CMR
Hu [19]	China	BM-MNCs	22	14	60.45 ± 11.4	60.62 ± 10.85	86	64	53.8 ± 11.5	57.1 ± 11.6	5	Echo/SPECT
Huang [20]	China	BM-MNCs	79	25	58.55 ± 8.72	58.8 ± 8.4	91	88	43.65 ± 5.21	43.5 ± 3.5	1–30	Echo/SPECT
Huikuri [21]	Finland	BM-MNCs	40	40	60 ± 10	59 ± 10	90	85	59 ± 11	62 ± 12	2–6	Echo/LV angiography
Janssens [22]	Belgium	BM-MNCs	33	34	55.8 ± 11	57.9 ± 10	82	82	48.5 ± 7.2	46.9 ± 8.2	1	Echo/CMR
Meluzin [23]	Czech	BM-MNCs	40	20	54 ± 2	55 ± 2	92	90	40.5 ± 8.94	40 ± 8.94	3–8	Echo/SPECT
Meyer [24]	Germany	BM-MNCs	30	30	53.4 ± 14.8	59.2 ± 13.5	67	73	50 ± 10	51.3 ± 9.3	4.8 ± 1.3	CMR
Nicolau [25]	Brazil	BM-MNCs	66	55	59.23 ± 9.44	58.72 ± 9.3	80	82	44.63 ± 10.74	42.23 ± 10.33	6–9	CMR
Nogueira [26]	Brazil	BM-MNCs	24	6	57.2 ± 10.9	57.2 ± 10.8	71	67	48.41 ± 8.28	47.59 ± 14.31	4–7.5	Echo
Piepoli [27]	Italy	BM-MNCs	19	19	63.1 ± 2.4	67 ± 2.7	68.4	68.4	38.9 ± 1.3	38.4 ± 1.5	4–7	Echo/SPECT
Plewka [28]	Poland	BM-MNCs	40	20	56 ± 9	56 ± 9	67	75	35 ± 6	33 ± 7	7	Echo/SPECT
San Roman [29]	Spain	BM-MNCs	30	31	54 ± 11	57 ± 11	97	90	49 ± 8	47 ± 8	3–5	CMR/LV angiography
Skalicka [30]	Czech	BM-MNCs	17	10	61 ± 14	54 ± 10	71	100	39.2 ± 9.2	39.4 ± 5.6	4–11	Echo/SPECT
Sürder [31]	Switzerland	BM-MNCs	128	67	58.44 ± 10.92	56 ± 10.74	84	84	36.4 ± 8.92	40 ± 9.9	5–28	CMR
Traverse [32]	USA	BM-MNCs	58	29	57.6 ± 11	54.6 ± 11	79	90	48.7 ± 12	45.3 ± 9.9	14–21	CMR
Traverse [33]	USA	BM-MNCs	30	10	52.5 ± 15.56	57.5 ± 3.7	83	60	49 ± 9.5	48.6 ± 8.5	3–10	CMR/Echo
Traverse [34]	USA	BM-MNCs	58	27	55.9 ± 11	56.4 ± 10.4	88	86	45.9 ± 9.4	46.9 ± 8.7	3–7	CMR
Turan [35]	Germany	BM-MNCs	42	20	61 ± 15	60 ± 11	67	70	43 ± 10	45 ± 10	7	Left Ventriculography
Wöhrle [36]	Germany	BM-MNCs	29	13	61 ± 8.1	61.1 ± 9.3	90	62	53.5 ± 9.3	55.7 ± 9.4	5–7	CMR
Wollert [37]	Germany	BM-MNCs	127	26	55.46 ± 9.83	55 ± 9	85	92	44.3 ± 8.48	47.8 ± 6.7	7.1 ± 2.6	CMR
Yao [38]	China	BM-MNCs	27	12	51.7 ± 6.4	52.7 ± 7.8	81	92	33.2 ± 3.9	32.3 ± 2	3d–3 m	CMR
Chullikana [39]	India	MSCs	10	10	47.31 ± 12.1	47.79 ± 6.48	100	80	43 ± 3.63	43.44 ± 4.4	2	Echo
Gao [40]	China	MSCs	21	22	55.0 ± 1.6	58.6 ± 2.5	100	86	50.2 ± 6.87	51.2 ± 7	17.1 ± 0.6	Echo
Gao [41]	China	MSCs	58	58	57.3 ± 1.3	56.7 ± 1.7	95	88	52 ± 0.9	51.1 ± 1	5–7	Echo
Hare [8]	USA	MSCs	34	19	59 ± 12.3	55.1 ± 10.2	79	82	48.7 ± 9.6	50.4 ± 10.6	–	Echo
Houtgraaf [42]	Netherlands	MSCs	9	4	61 ± 2.1	55 ± 7.5	78	100	52.1 ± 2.5	52 ± 10	1	SPECT
Kim [43]	South Korea	MSCs	14	12	55.3 ± 8.6	57.8 ± 8.9	100	100	35.1 ± 4.5	37.4 ± 1.7	30 ± 1.3	Echo/SPECT
Lee [44]	South Korea	MSCs	30	28	53.9 ± 10.5	54.2 ± 7.7	90	89	48.1 ± 8	51 ± 9.2	25 ± 2.4	Echo/SPECT
Penn [45]	USA	MSCs	19	6	56.8 ± 8.7	53 ± 8	63	83	47.9 ± 10.2	42.3 ± 6.4	3.3	Echo
Rodrigo [46]	Netherlands	MSCs	9	45	56 ± 8	61 ± 11	78	78	48 ± 2	45 ± 9	21 ± 3	Echo
Zhang (47)	China	MSCs	21	22	59.3 ± 9	58.6 ± 11	95	86	57.2 ± 10.2	53.7 ± 6.4	14 ± 9.5	Echo

**Table 2:** Assessment of credibility of subgroup difference based on ICEMAN

Variable	Q1	Q2	Q3	Q4	Q5	Q6	Q7	Q8	Overall interpretation	
LVEF	Completely between	NA	Large	Definitely yes	Chance a very likely explanation (0.1)	Definitely yes	Definitely yes	NA	Maximum usually moderate	Likely effect modification. Use separate effects for each subgroup but note remaining uncertainty
LVEF (sensitivity analysis)	Completely between	NA	Rather small or unclear	Definitely yes	Chance a very likely explanation (0.06)	Definitely yes	Definitely yes	NA	Maximum usually low	Likely no effect modification. Use overall effect for each subgroup but note remaining uncertainty
LVESV	Completely between	NA	Rather large	Definitely no	Chance a very likely explanation (0.92)	Definitely yes	Definitely yes	NA	Maximum usually low	Likely no effect modification. Use overall effect for each subgroup but note remaining uncertainty
LVESV (sensitivity analysis)	Completely between	NA	Very small	Definitely yes	Chance a very likely explanation (0.15)	Definitely yes	Definitely yes	NA	Maximum usually low	Likely no effect modification. Use overall effect for each subgroup but note remaining uncertainty
LVEDV	Completely between	NA	Rather large	Definitely no	Chance a very likely explanation (0.84)	Definitely yes	Definitely yes	NA	Maximum usually low	Likely no effect modification. Use overall effect for each subgroup but note remaining uncertainty
LVEDV (sensitivity analysis)	Completely between	NA	Very small	Definitely yes	Chance may not explain (0.007)	Definitely yes	Definitely yes	NA	Maximum usually moderate	Likely effect modification. Use separate effects for each subgroup but note remaining uncertainty
Hospitalization	Completely between	NA	Rather large	Definitely no	Chance a very likely explanation (0.26)	Definitely yes	Definitely yes	NA	Maximum usually low	Likely no effect modification. Use overall effect for each subgroup but note remaining uncertainty

Q1, Is the analysis of effect modification based on comparison within rather than between trials? Q2, for within-trial comparisons, is the effect modification similar from trial to trial? Q3, for between-trial comparisons, is the number of trials large? Q4, Was the direction of the effect modification correctly hypothesized priori? Q5, does a test for interaction suggest that chance is an unlikely explanation of the apparent effect modification? Q6, Did the authors test only a small number of effect modifiers? Q7, Did the authors use a random effects model? Q8, If the effect modifier is a continuous variable, were arbitrary cut points avoided?

## Supplementary information

**Additional file 1.** Summary of the risk of bias in the included studies.

**Additional file 2.** Forest plot of the effect sizes of changes in LVEF from baseline during the short-term follow-up (4-6 months) measured by echocardiography in acute MI patients who received an intracoronary injection of either BM-MNCs or MSCs before 11 days after diagnosis of acute MI compared to the control group who received standard therapy with or without placebo injection.

**Additional file 3.** Forest plot of LVEDV changes measured by echocardiography in acute MI patients receiving either standard therapy (with or without placebo injection) or autologous intracoronary injection of stem cells before 11 days of diagnosis based on the type of cell (BM-MNCs or MSCs).

**Additional file 4.** Forest plot of comparison of changes in LVESV over the follow-up period measured by echocardiography in patients with acute MI who received intracoronary injection of stem cells based on the type of cells (BM-MNCs or MSCs) before 11 days after diagnosis of MI compared to the control group.

## Data Availability

The data underlying this article will be shared on reasonable request to the corresponding author.

## Abbreviations

AMI: Acute myocardial infarction
HF: Heart failure
CHF: Congestive heart failure
BM-MNC: Bone marrow mononuclear cell
MSC: mesenchymal stem cell
IL-1: interleukin-1
IL-6: interleukin-6
TNF-α: tumor necrosis factor-α
PSC: progenitor stem cell
LVEF: left ventricular ejection fraction
LVEDV: left ventricular end-diastolic volume
LVESV: left ventricular end-systolic volume
STEMI: ST elevation myocardial infarction
SD: standard deviation
RR: risk ratio
CI: confidence interval
WMD: weighted mean difference
ICEMAN: instrument for assessing the credibility of effect modification of analyses
CMR: cardiac magnetic resonance
SPECT: single-photon emission computed tomography
PCI: percutaneous coronary intervention
