# Supplementary figures and images for: Comparing the effect of bone marrow mono-nuclear cells with mesenchymal stem cells after acute myocardial infarction on improvement of left ventricular function: a meta-analysis of clinical trials
Source: Stem Cell Res Ther. 2022 May 16;13:203. doi: 10.1186/s13287-022-02883-3 (PMC9109324; doi:10.1186/s13287-022-02883-3)

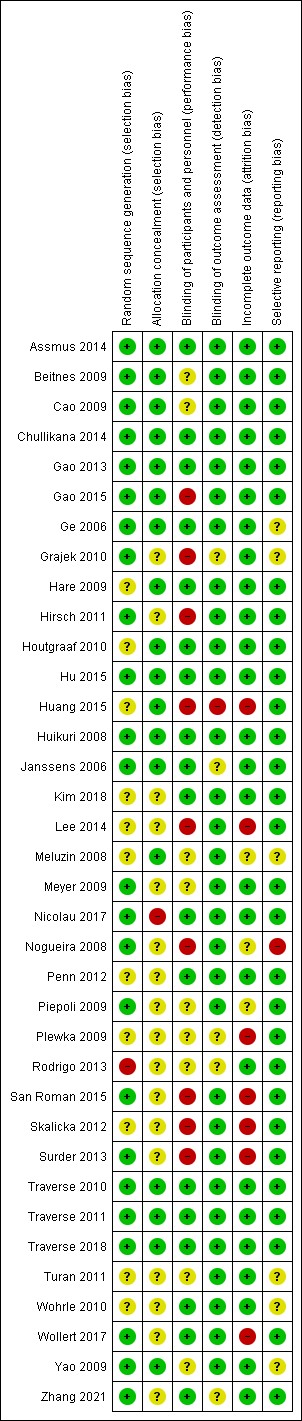

Supplement: Supplementary file 1 — Additional file 1. Summary of the risk of bias in the included studies. [file 13287_2022_2883_MOESM1_ESM.jpg]

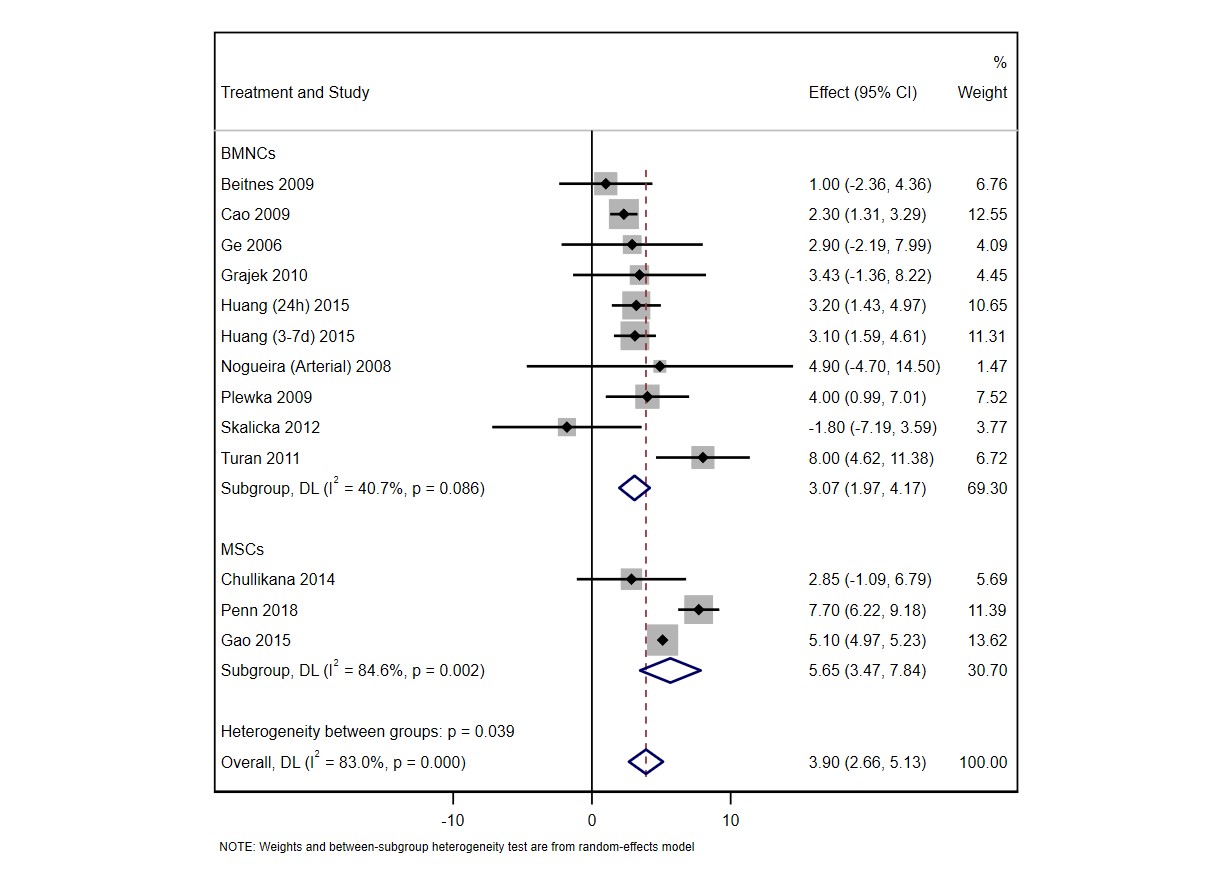

Supplement: Supplementary file 2 — Additional file 2. Forest plot of the effect sizes of changes in LVEF from baseline during the short-term follow-up (4-6 months) measured by echocardiography in acute MI patients who received an intracoronary injection of either BM-MNCs or MSCs before 11 days after diagnosis of acute MI compared to the control group who received standard therapy with or without placebo injection. [file 13287_2022_2883_MOESM2_ESM.jpg]

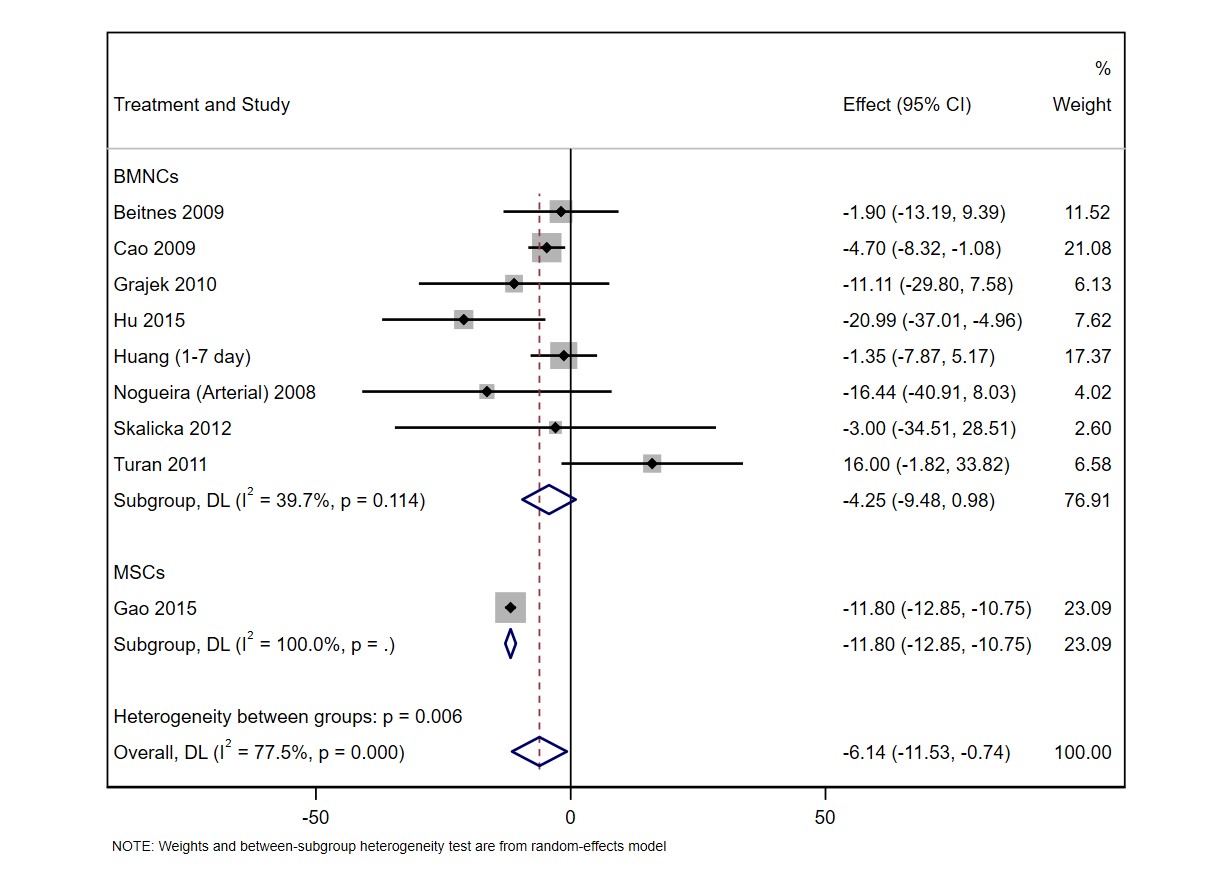

Supplement: Supplementary file 3 — Additional file 3. Forest plot of LVEDV changes measured by echocardiography in acute MI patients receiving either standard therapy (with or without placebo injection) or autologous intracoronary injection of stem cells before 11 days of diagnosis based on the type of cell (BM-MNCs or MSCs). [file 13287_2022_2883_MOESM3_ESM.jpg]

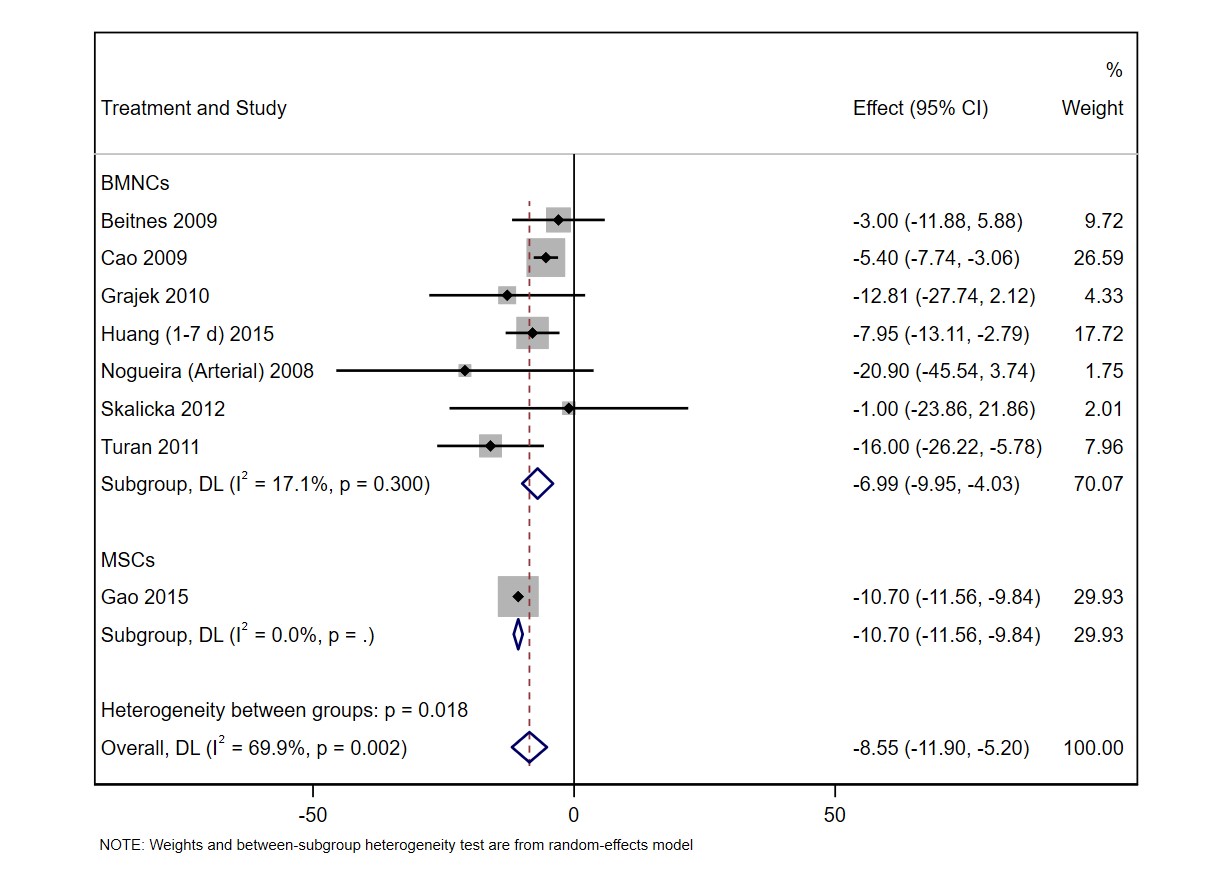

Supplement: Supplementary file 4 — Additional file 4. Forest plot of comparison of changes in LVESV over the follow-up period measured by echocardiography in patients with acute MI who received intracoronary injection of stem cells based on the type of cells (BM-MNCs or MSCs) before 11 days after diagnosis of MI compared to the control group. [file 13287_2022_2883_MOESM4_ESM.jpg]
